# Real-World Utilization and Patient Acceptance of Telemedicine in Otorhinolaryngology: A Single-Center Observational Study

**DOI:** 10.3390/jcm15187203

**Published:** 2026-09-16

**Authors:** Kathrin Gerstacker, Sarah Riemann, Iva Speck, Andreas Knopf

**Affiliations:** 1 Department of Oto-Rhino-Laryngology, Medical Center, University of Freiburg, Killianstr. 5, 79106 Freiburg, Germany; 2 Department of Nuclear Medicine, Medical Center, University of Freiburg, Hugstetterstr. 53, 79106 Freiburg, Germany

**Keywords:** telemedicine, video consultation, ENT, outpatient care, real-life

## Abstract

**Background**: Telemedicine may improve access to care and streamline patient pathways, but real-world uptake in otorhinolaryngology remains uncertain. This single-center study evaluated an online appointment module with an option for video consultation at ENT University Hospital Freiburg and compared real-world utilization with patients’ hypothetical willingness to use telemedicine. **Materials and Methods**: The online module was implemented from March to June 2021. We recorded symptom category, age, gender, distance to the clinic, insurance status, type of consultation requested, and preference for video versus face-to-face consultation. Video consultations were evaluated separately in March and April. In addition, an independent survey was conducted in February 2021 among patients presenting with otological symptoms to assess hypothetical willingness to substitute an equivalent telemedical consultation for an in-person visit. **Results**: Of 7435 outpatient contacts, 507 (6.8%) used the online appointment module. Among these, 35 (6.9%) requested a video consultation and 472 (93.1%) requested a face-to-face appointment; video requests therefore represented 0.47% of all outpatient contacts. By contrast, 35 of 55 survey respondents (63.6%) stated that they would forgo an in-person visit if equivalent telemedical care were available. In March and April, 29 video consultations were scheduled or requested after telephone counseling, of which 11 were completed. Nine of the 11 completed video consultations (81.8%) did not require a subsequent in-person visit, whereas two (18.2%) required additional audiometry or nasal endoscopy. Because only 11 video consultations were completed, these findings are descriptive and exploratory. **Conclusions**: Hypothetical willingness to use telemedicine was substantially higher than real-world video consultation utilization. This gap indicates that stated acceptance does not necessarily translate into actual use and may reflect clinical suitability, awareness, organizational or technical barriers, or patient preference. ENT departments considering telemedicine should therefore use targeted patient selection and pre-consultation triage, particularly for follow-up visits, counseling, and second opinions that do not require immediate examination or diagnostic testing. Generalizability is limited by the single-center design, the short observation period, the small number of completed video consultations, and the COVID-19 pandemic context.

## 1. Introduction

Telemedicine has become increasingly relevant in outpatient care as regulatory changes and the COVID-19 pandemic accelerated the use of digital healthcare. In Germany, the E-Health Act, the relaxation of restrictions on remote consultation, and subsequent reimbursement developments facilitated the implementation of telemedical services [1]. Telemedicine encompasses medical care delivered across physical and, in asynchronous settings, temporal distance [2]. In otorhinolaryngology, potential benefits include improved accessibility, reduced travel and contact, and continuity of care when direct access is limited [2,3]. During the COVID-19 pandemic, remote consultation also offered a means of reducing potentially infectious contacts [4,5].

Despite these potential advantages, telemedicine in ENT has important limitations. Many diagnostic pathways depend on physical examination or technical procedures such as otoscopy, audiometry, endoscopy, sonography, or palpation, which cannot be fully reproduced in a conventional video consultation [2,3]. Previous studies have nevertheless reported favorable experiences with telemedicine, particularly for selected follow-up visits and postoperative consultations [6,7,8]. Patient acceptance has also often been reported as high, whereas actual utilization may remain low [1]. Thus, hypothetical willingness to use telemedicine may not reliably predict real-world adoption.

The present study therefore evaluated three related but distinct components: (a) real-world use of an asynchronous online appointment module, (b) real-world use and feasibility of synchronous video consultations, and (c) an independent pre-implementation survey of hypothetical willingness to use telemedicine. The survey participants were not the same cohort as the patients subsequently using the online module. The primary aim was to compare stated willingness with actual utilization and to identify potential opportunities and limitations of telemedicine in an ENT outpatient setting. Because the study was conducted at a single tertiary center over a four-month period during the COVID-19 pandemic, the findings must be interpreted in this specific institutional and pandemic context.

## 2. Materials and Methods

### 2.1. Study Design

This prospective single-center observational study evaluated a newly implemented telemedicine service at the ENT University Hospital Freiburg. The study comprised three distinct components: (a) asynchronous online appointment requests from March to June 2021, (b) synchronous video consultations, evaluated in detail in March and April 2021, and (c) an independent pre-implementation survey on hypothetical willingness to use telemedicine conducted in February 2021. Survey participants were not the same cohort as patients subsequently using the online appointment module. The four-month implementation period and single-center setting were predefined characteristics of this real-world evaluation.

(a) Online appointment scheduling and (b) Video consultation

The study distinguished between (a) asynchronous online appointment requests and (b) synchronous video consultations. According to Hagge et al., asynchronous telemedicine separates communication in time, whereas synchronous telemedicine involves real-time doctor-patient interaction [2]. In the present study, the online module allowed patients to submit an appointment request, select a symptom category and, optionally, provide a written free-text description. Video consultation was defined as real-time doctor-patient contact by video.

All ENT patients who submitted an appointment request through the online module between March and June 2021 were included in the analysis of real-world digital utilization. Video consultations were evaluated separately in March and April. Following Xie et al., a completed video consultation was considered successful when no subsequent in-person visit was required [8,9]. During these two months, patients who initially requested a face-to-face appointment through the online module were contacted by an ENT specialist and informed about the option of video consultation as an initial contact. Because only 11 video consultations were ultimately completed, analyses of this subgroup were considered descriptive and exploratory.

(c) Survey on willingness to use telemedicine

From 15 February to 28 February 2021, before implementation of the online module, an independent survey was conducted in the general ENT outpatient clinic. The survey targeted patients presenting with otological symptoms and assessed whether they would be willing to forgo their current in-person ENT visit if equivalent telemedical treatment were available. The survey cohort was separate from the subsequent March–June 2021 online-module cohort. During the survey period, 828 patients with various ENT complaints attended the outpatient clinic. Patients were eligible for the survey if they presented with otological symptoms and were legally competent to provide informed consent. Patients presenting with non-otological complaints were excluded. Seventy-three patients (8.8% of all outpatient presentations) fulfilled the symptom-related eligibility criterion, and 55 completed the questionnaire. The following questions were asked:

1. Would you be willing to give up your current ENT outpatient visit if we could offer you equivalent treatment via telemedicine?
  a. Yes
  b. No
2. If no: Which of the following reasons do you consider to be the main reason against telemedical treatment?
  a. I do not have sufficient internet or do not own a smartphone, computer, or tablet.
  b. I do not have sufficient knowledge of technology, the internet, etc.
  c. I have concerns about data protection.
  d. I do not believe that the quality of treatment is the same as with a conventional visit to the doctor.
  e. I would like to speak to a doctor in person and feel better treated in a clinic/doctor’s surgery.
  f. Other reason with free text option

### 2.2. Study Population and Collected Data

(a) Online appointment scheduling and (b) Video consultation

For the online appointment module, patient data included age, gender, insurance status (statutory versus private), and distance from the place of residence to the clinic. Age was described using the prespecified categories <50 and ≥50 years. The 50-year threshold was a pragmatic descriptive cut-off used to compare younger and older users of the digital service and was not intended as a clinically validated threshold. The following information was also collected: type of consultation requested (specialized versus general ENT consultation), preferred first contact (video versus face-to-face consultation), and symptom category (nose/sinuses, ear/hearing, throat/oral cavity, voice/swelling, sleep disorders, salivary glands, other, and an optional free-text description).

Secondly, the implementation of the (b) video consultation was examined in the months of March and April. For this purpose, patients who had submitted an online telemedicine request were contacted by telephone by a specialist and informed in more detail about the possibility of a video consultation and, depending on the patient’s wishes, scheduled for a video consultation. We investigated the success of the video consultation in terms of the need for a personal reappointment following the video consultation versus a completed consultation, as well as the reasons for this and the subject of the complaint.

(c) Survey on willingness to use telemedicine

For the third component, hypothetical willingness to use telemedicine was assessed prospectively during the two-week pre-implementation survey. Participants were asked (A) whether they would forgo the current in-person visit if equivalent telemedical treatment were available and, if not, (B) their main reason for declining telemedicine. Gender, age, insurance status, and place of residence were also recorded. This survey was independent of the subsequent online appointment and video-consultation cohorts.

For the survey, patients were eligible if they presented with otological symptoms and were legally competent to provide informed consent. Patients presenting with non-otological complaints were excluded. During the two-week recruitment period, 828 patients with various ENT complaints attended the outpatient clinic. Of these, 73 patients (8.8%) presented with otological symptoms and were therefore eligible for the survey; the remaining 755 patients presented with non-otological complaints and were not eligible. Of the 73 eligible patients, 55 participated, corresponding to a response rate of 75.3% among eligible patients.

The study is based on statistical data from the ENT University Clinic Freiburg, the patient master data, the recorded telemedicine inquiries, and the video call recordings.

All procedures performed in studies involving human participants were in accordance with the ethical standards of the institutional and/or national research committee and with the 1964 Helsinki Declaration and its later amendments or comparable ethical standards.

### 2.3. Statistical Analysis

Statistical analysis was performed using R version 4.0.0 (R Foundation for Statistical Computing, Vienna, Austria). Categorical variables are presented as absolute numbers and percentages and were compared using the chi-square test or Fisher’s exact test, as appropriate. Continuous variables are reported as mean ± standard deviation and, where appropriate, median and range. Comparisons of continuous variables between two groups were performed using an unpaired Student’s *t*-test. Age was additionally categorized as <50 and ≥50 years for descriptive subgroup analyses. The cut-off of 50 years was chosen pragmatically and does not represent a clinically established threshold. Given the small number of completed video consultations (*n* = 11), analyses relating to this subgroup were considered descriptive and exploratory. A two-sided *p*-value ≤ 0.05 was considered statistically significant.

### 2.4. Ethical Approval and Patient Consent

The present prospective study was conducted in accordance with the guidelines of the Declaration of Helsinki (Washington, World Medical Association, 2013), was approved by the ethics committee of the University of Freiburg (20-1332) and registered at the German clinical trials register (DRKS00021782, approval Date: 10 September 2020). Included participants gave written informed consent before inclusion in the present study.

## 3. Results

(a) Online appointment scheduling and (b) Video consultation

The overall results are summarized in Table 1.

From March to June 2021, 507 of 7435 outpatient contacts (6.8%) used the asynchronous online appointment module. Of these 507 online requests, 35 patients (6.9%) requested a video consultation, whereas 472 (93.1%) requested a face-to-face appointment. Thus, requests for video consultation accounted for only 0.47% of all outpatient contacts during the study period. These figures describe real-world utilization and should be distinguished from the separate pre-implementation survey of hypothetical willingness.

### 3.1. Age of Patients

In total, 155 (30.63%) patients using the online appointment system were ≥50 years, and 8 (5.2%) of them asked for a video consultation; 351 (69.37%) patients were ≤50 years, and 27 (7.7%) of these asked for a video consultation.

### 3.2. Type of Insurance Status of the Patients

A total of 459 (90.5%) patients had public insurance and 48 (9.5%) patients had private insurance, which corresponds approximately to the insurance distribution of the general population [10]. Insurance status is comparable in the age groups ≥ 50 years (90.9% with public insurance and 9.1% with private insurance) and <50 years (89.7% with public insurance and 10.3 with private insurance).

### 3.3. Distance from the Patient’s Place of Residence to the ENT University Hospital

The distance between the patient’s place of residence and the ENT University Hospital Freiburg, i.e., corresponding to the patient’s travel distance, was at least 1 km and a maximum of 219 km, with an average of 36.91 (standard deviation of 32.09 km). The age group ≥ 50 years had a minimum travel distance of 1 km and a maximum of 162 km, with an average of 43.98 km and a median of 40.0 (standard deviation of 32.09). For the age group < 50 years, a distance of at least 1 km and a maximum of 219 km was recorded, with an average of 33.49 km and a median of 23.0 (31.183 standard deviation). Patients who made an online telemedicine request at the ENT University Hospital Freiburg had a slightly longer travel distance in the age group > 50 years, with an average of 43.98 km, compared to the younger age group of < 50 years with an average of 33.49 km.

### 3.4. Selection of the Symptom Category

Patients could choose between eight different symptom categories to categorize their complaints. Of the 507 telemedicine inquiries, 154 (30.4%) were categorized as nose/sinuses, 189 (37.3%) as ear/hearing symptoms, 80 (15.8%) as throat/oral cavity, 21 (4.1%) as voice/swelling, 14 (2.8%) as sleep disturbance, 13 (2.6%) as salivary glands and 36 (7.1%) as other symptoms.

**Figure 1:**
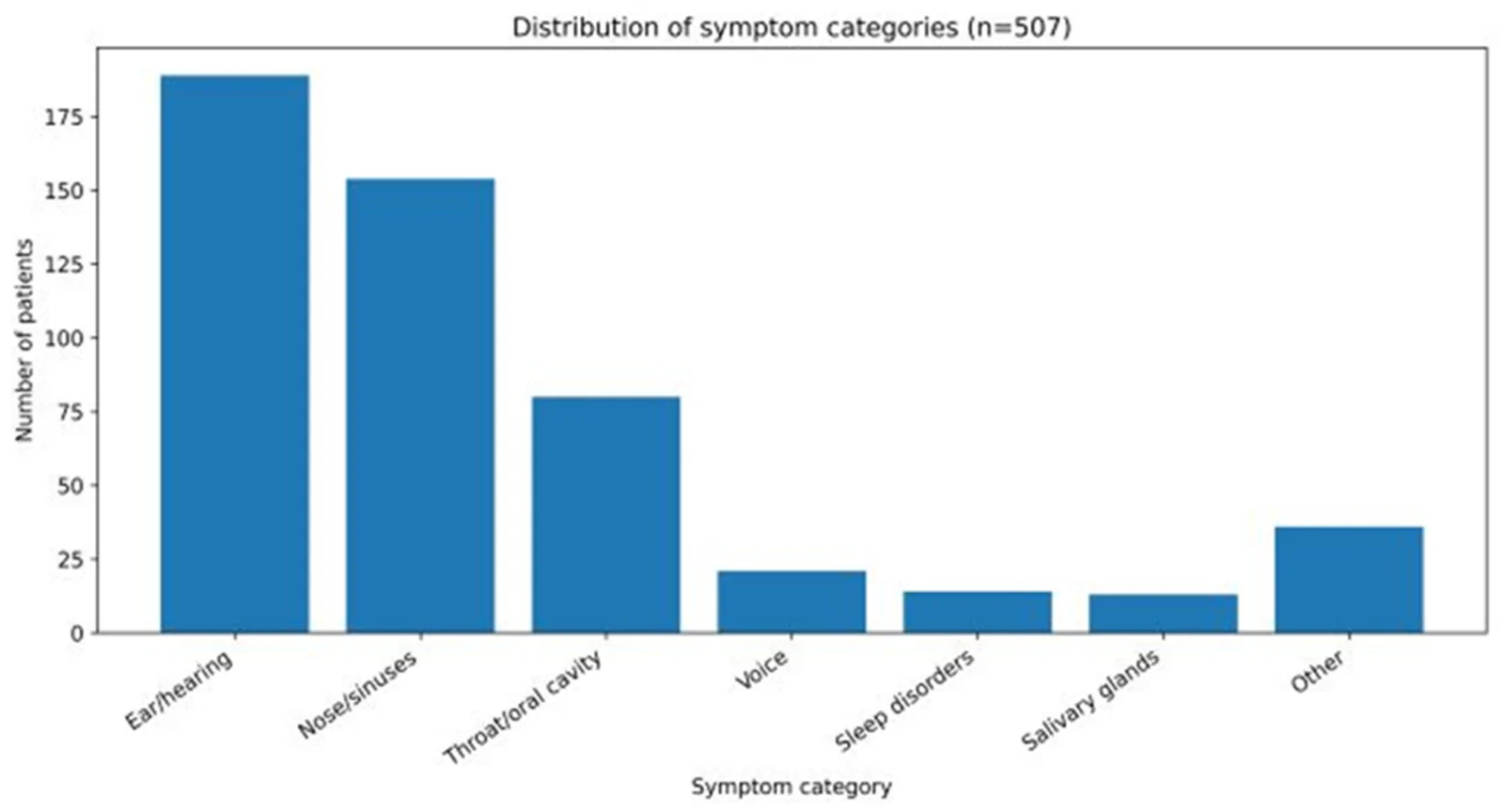
Symptoms selected by patients asking for an online appointment.

Divided into the two age groups (≥50 years and ≤50 years), these symptom categories, nose/sinus and ear/hearing, predominated (Figure 1).

### 3.5. Description of Complaints via the Online Portal

Patients had the opportunity to freely formulate their complaints in writing in advance through the online telemedicine inquiry. Only 2.8% of all patients used this option. The age group < 50 years was more strongly represented, with 3.4% compared to 1.3% of the age group ≥ 50 years.

### 3.6. Preference for the Type of Consultation

Patients were able to choose between the categories of a general ENT consultation and a special ENT consultation. 385 (75.9%) patients indicated a preference for a general consultation and 122 (24.1%) patients indicated a preference for a special consultation when making an appointment online. In the age group ≥ 50 years, 50 (32.3%) requested a special consultation. In the age group < 50 years, 72 (20.5%) patients requested a special consultation.

### 3.7. Timeline of the Online Telemedicine Inquiries

**Figure 2:**
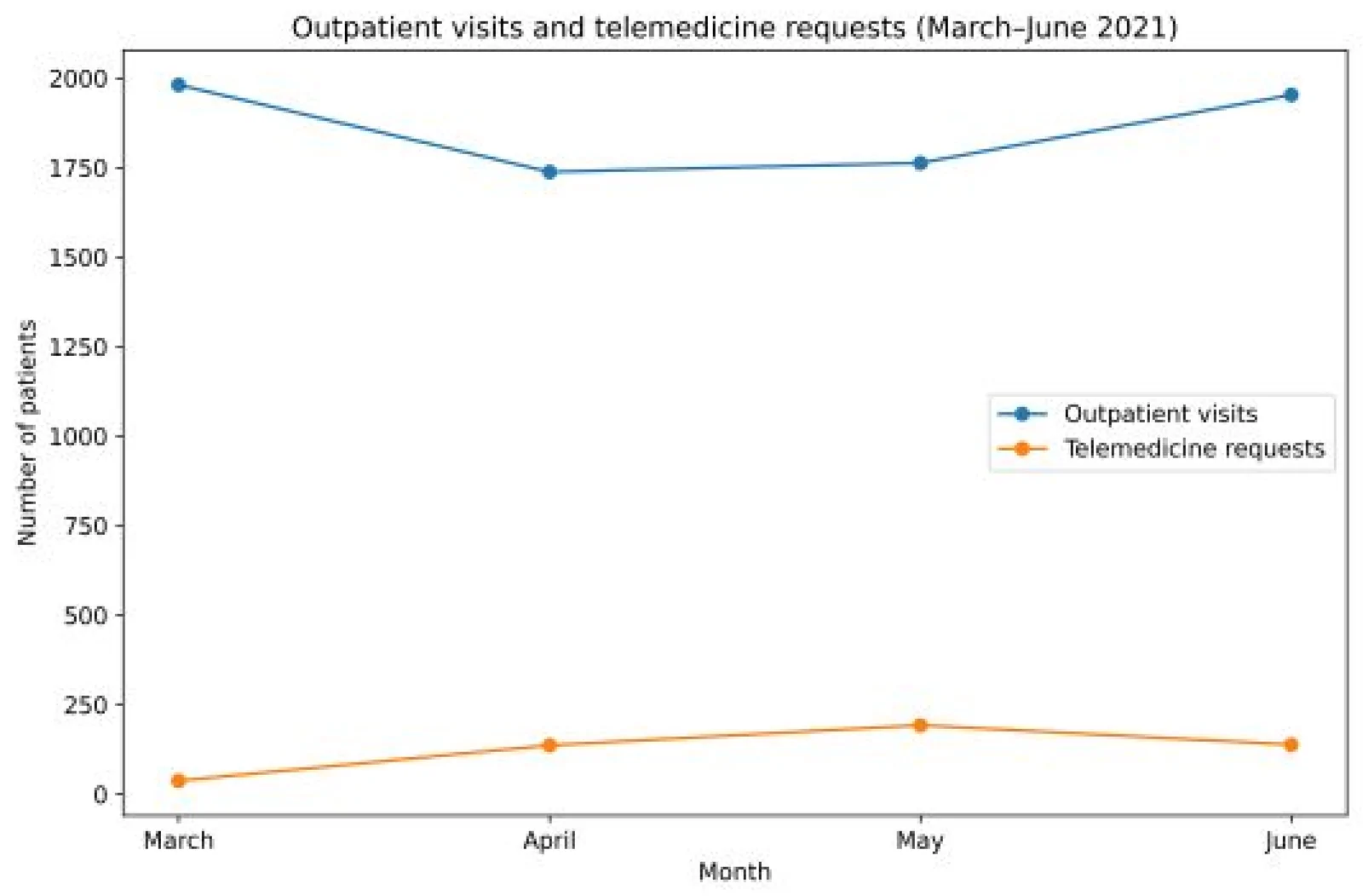
Consultations in the open ENT outpatient clinic.

During the four-month study period, 38 online requests were submitted in March 2021, 137 in April, 193 in May, and 139 in June. The number of requests increased after the initial implementation phase, peaked in May, and declined in June. In-person outpatient contacts were 1981 in March, 1738 in April, 1763 in May, and 1953 in June (Figure 2). Given the short observation period, the decline after May cannot be interpreted as evidence that future demand would not increase.

### 3.8. Results of the Implementation of the Video Consultation

In March and April, 175 patients used the online appointment module. Ten (5.71%) initially requested a video consultation. Of the remaining 165 patients who initially requested a face-to-face appointment, 19 (11.52%) opted for video consultation after telephone counseling by an ENT specialist, resulting in 29 planned or requested video consultations. Eleven video consultations were ultimately completed. Six requests were redirected to prompt in-person care by the physician because of emergency symptoms, suspected tumor, or the need for polygraphy adjustment. Twelve scheduled video consultations were not completed because no suitable appointment could be found, the patient canceled, or no reason was documented. Of the 11 completed video consultations, nine (81.8%) required no subsequent in-person visit and therefore met the predefined success criterion. Two (18.2%) required additional face-to-face assessment: one for audiometry in suspected sudden hearing loss and one for nasal endoscopy in unclear nasal obstruction. Six of the 11 completed consultations (54.5%) concerned second opinions regarding a proposed operation, and three concerned tinnitus. Consultation type and successful completion are reported separately because a second-opinion consultation does not, by itself, define success. One consultation was complicated by a language barrier but was still completed. Owing to the small number of completed video consultations, these findings are descriptive and exploratory.

### 3.9. Survey on Willingness to Use Telemedicine

**Figure 3:**
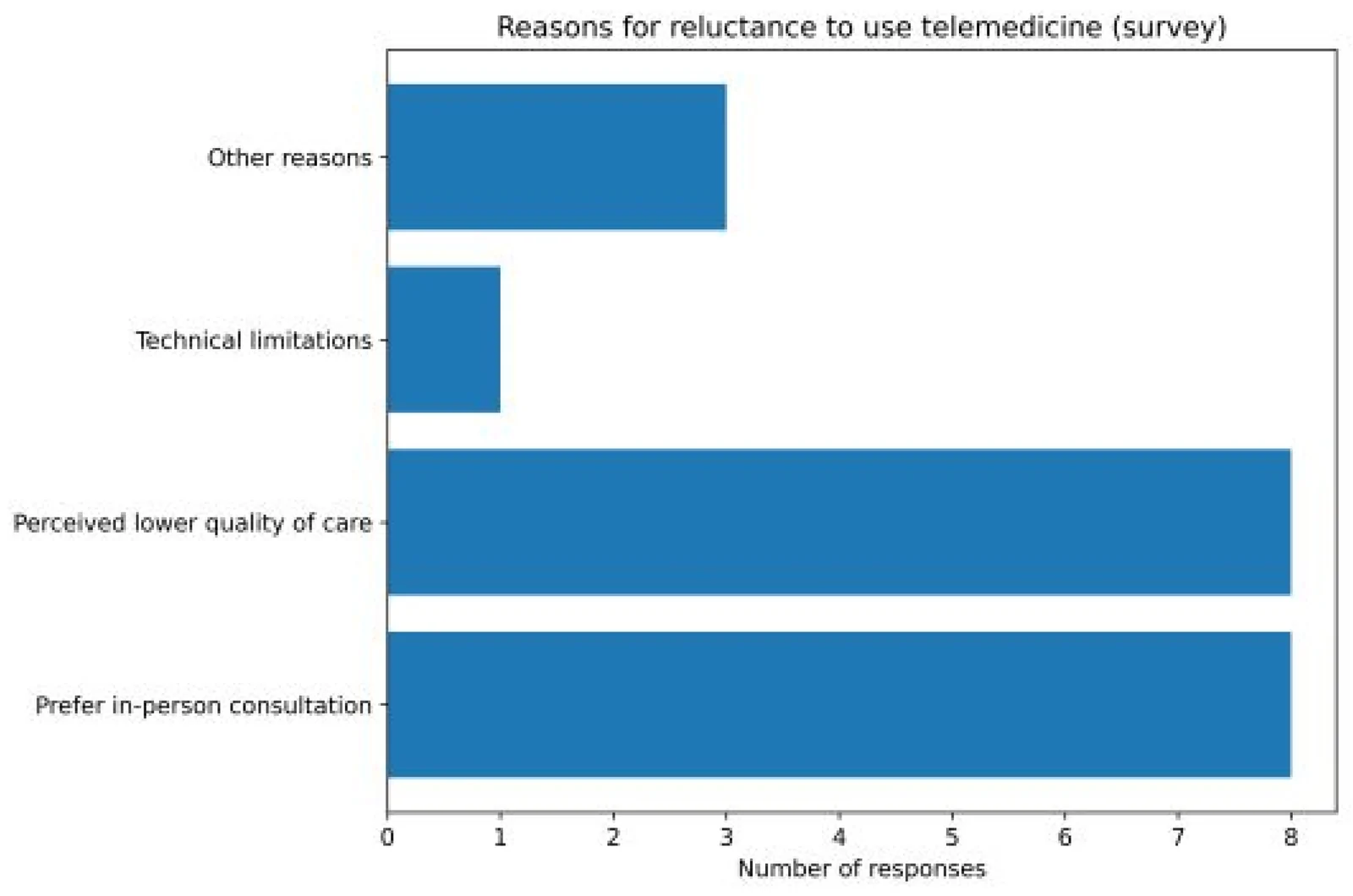
Reasons given for reservations about telemedical care.

During the survey period from 15 February to 28 February 2021, a total of 828 patients presented to the general ENT outpatient clinic with various ENT complaints. Of these, 73 patients (8.8%) presented with otological symptoms and were therefore eligible for the survey; the remaining 755 patients presented with non-otological complaints and were not eligible. Of the 73 eligible patients, 55 completed the questionnaire, corresponding to a response rate of 75.3%. Of these 55 participants, 35 (63.6%) stated that they would have forgone the in-person ENT outpatient visit if equivalent treatment via telemedicine had been available, whereas 20 (36.4%) preferred an in-person consultation. Of these 20 patients, 19 provided a reason for declining telemedical care; one participant did not specify a reason. Among the 19 respondents who provided a reason, eight cited concerns regarding equivalent treatment quality and/or a preference for direct personal contact with a physician. Two participants provided additional free-text reasons emphasizing the importance of face-to-face examination and communication, and one participant reported insufficient internet access or lack of an appropriate device (Figure 3).

## 4. Discussion

The principal finding of this study is the marked discrepancy between hypothetical willingness to use telemedicine and actual utilization in routine ENT care. Before implementation, 35 of 55 survey respondents (63.6%) stated that they would forgo an in-person visit if equivalent telemedical care were available. During the subsequent four-month implementation period, however, only 35 of 507 online requests selected video consultation, corresponding to 6.9% of online requests and 0.47% of all outpatient contacts. This finding suggests that stated willingness in a hypothetical survey does not necessarily translate into real-world use. The present study was not designed to determine the causes of this gap. Limited awareness, technical or organizational barriers, established care-seeking habits, and the perceived need for physical examination are plausible explanations, but they should be regarded as hypotheses rather than conclusions from our data. The fact that 19 of 165 patients who initially requested face-to-face care changed to video consultation after telephone counseling suggests that active information may influence uptake, although the small numbers preclude firm conclusions [11].

The distinction between asynchronous and synchronous telemedicine is also relevant. The online appointment module was used by 507 patients, whereas only 35 requested synchronous video consultation. Moreover, only 2.8% of users made use of the optional free-text field to describe their complaints. Thus, engagement with the asynchronous module primarily consisted of structured appointment requests rather than detailed written clinical communication. These findings indicate that use of a digital access channel should not be equated with willingness to receive clinical care by video. Future implementations should evaluate the different functions of asynchronous scheduling, written symptom collection, and synchronous consultation separately.

ENT-specific diagnostic requirements may partly explain the limited suitability of video consultation [12,13,14]. Otoscopy, audiometry, nasal endoscopy, sonography, palpation, and office-based procedures cannot be fully replaced by standard video contact [2,3,15]. This was reflected in the completed video consultations: nine of 11 (81.8%) were completed without a subsequent in-person visit, whereas two required additional audiometry or nasal endoscopy. Six of the 11 completed consultations were second-opinion consultations, but consultation type must be distinguished from the predefined success criterion of avoiding subsequent in-person assessment. Given the very small number of completed video consultations, these observations are exploratory and do not support robust subgroup conclusions. Nevertheless, they suggest that follow-up visits, counseling, postoperative review and selected second opinions may be more appropriate targets than undifferentiated first-contact care [6,7,8,16].

The findings also have practical implications for implementation. Rather than offering video consultation indiscriminately, ENT departments could use pre-consultation triage to identify patients whose clinical question can reasonably be addressed without immediate physical examination or diagnostic testing [15,16]. Potential target groups include selected follow-up visits, postoperative reviews, counseling encounters such as tinnitus consultations, and second opinions based on already available diagnostic information. Active patient information may be important, because additional patients accepted video consultation after telephone counseling in this study [17,18,19]. At the organizational level, simple technical access, clear scheduling pathways, defined criteria for conversion to in-person care, and integration into existing clinical workflows may reduce barriers. Broader digital-health literature similarly emphasizes that successful adoption of new technologies depends not only on user acceptance but also on training, accessibility, standards, reimbursement, data structures, and integration into clinical workflows [20,21]. These parallels support interpreting low uptake as a potential implementation issue rather than solely as a measure of patient preference.

Several limitations should be considered. First, this was a single-center study conducted at a tertiary university ENT department over only four months, which limits generalizability and does not permit conclusions about longer-term adoption. Second, only 11 video consultations were completed; analyses of this subgroup are therefore descriptive and exploratory, and the absence of statistical significance must not be interpreted as evidence of no association. Third, the study took place during the COVID-19 pandemic. Infection concerns could have increased interest in remote care, whereas mask mandates, testing requirements, changing access rules, altered healthcare-seeking behavior, and the perceived need for in-person examination could have influenced preferences in either direction [9,20,22]. Outpatient volumes from March to June 2019 (7822 presentations) were broadly comparable with those in 2021 (7435 presentations), but similar overall volumes cannot exclude pandemic-related changes in patient behavior or case mix. The 2019 comparison therefore does not establish that the 2021 findings were independent of the pandemic. Fourth, the pre-implementation survey and the subsequent online-module cohort were separate populations, so the 63.6% hypothetical willingness rate cannot be interpreted as an expected utilization rate in the later cohort. Finally, although 73 of 828 outpatient contacts were classified as eligible for the otology-focused survey and 55 responded, the available study documentation does not provide sufficiently detailed exclusion criteria to reconstruct why every non-participant was excluded. This limits assessment of recruitment-related selection bias.

## 5. Conclusions

In this single-center real-world study, hypothetical willingness to use telemedicine was substantially higher than actual utilization of video consultation. This discrepancy indicates that patient acceptance measured in a survey should not be equated with real-world adoption. Video consultation may be most useful for selected ENT encounters that do not require immediate physical examination or diagnostic testing, including follow-up visits, counseling, and second opinions. Implementation should therefore combine pre-consultation triage, active patient education, simple technical access, and clear pathways for conversion to in-person care when necessary. Because the study was conducted over a short period during the COVID-19 pandemic and only 11 video consultations were completed, these findings should be considered exploratory and require confirmation in larger, multicenter prospective studies.

## Figures and Tables

**Table 1:** Characteristics of included participants.

Variable	Total	Age = 50 Years	Age < 50 Years
Gender	male 255, female 240, missing 12	male 79, female 72	male 176, female 168
Age (years)	*n* = 507	*n* = 155	*n* = 351
Distance to clinic (km)	range 1–219, mean 36.91	range 1–162, mean 43.98	range 1–219, mean 33.49
Insurance status	statutory 459, private 48	statutory 139, private 16	statutory 319, private 32
Type of consultation	general 385, specialized 122	general 105, specialized 50	general 279, specialized 72
Complaint description	yes 14, no 493	yes 2, no 153	yes 12, no 339
Preferred consultation	in person 472, video 35	in person 147, video 8	in person 324, video 27

Note: Age data were missing for one patient.

## Data Availability

The raw data supporting the conclusions of this article will not be made available by the authors because of ethical constraints.
